# Association of COVID-19 Infection With Survival After In-Hospital Cardiac Arrest Among US Adults

**DOI:** 10.1001/jamanetworkopen.2022.0752

**Published:** 2022-03-02

**Authors:** Saket Girotra, Maya L. Chan, Monique Anderson Starks, Matthew Churpek, Paul S. Chan

**Affiliations:** 1 Division of Cardiovascular Medicine, Department of Internal Medicine, University of Iowa Carver College of Medicine, Iowa City; 2 Center for Access Delivery Research and Evaluation, Iowa City Veterans Affairs Medical Center, Iowa City; 3 Pembroke Hill High School, Kansas City, MO; 4 Division of Cardiology, Duke University, Durham, NC; 5 Division of Allergy, Pulmonary, and Critical Care, Department of Medicine, University of Wisconsin, Madison; 6 Saint Luke’s Mid America Heart and Vascular Institute, University of Missouri, Kansas City

## Abstract

This cohort study examines the association of COVID-19 infection with survival outcomes of US adults after in-hospital cardiac arrest.

## Introduction

Early on in the COVID-19 pandemic, investigators reported poor survival rates (<3%) after in-hospital cardiac arrest (IHCA) among patients with COVID-19 infection in the US and China.^1,2,3^ These findings have prompted discussions regarding universal do-not-resuscitate orders for patients with COVID-19.^4^ However, these results were from single-center studies that comprised only 295 patients with COVID-19 in hospitals that were overwhelmed early during the pandemic. Whether the poor IHCA survival rate reported in earlier studies is broadly representative of patients with COVID-19 in US hospitals remains unknown. This study examined the association of COVID-19 infection with survival outcomes of US adults after IHCA.

## Methods

This cohort study was approved by the Saint Luke’s Hospital Institutional Review Board. The board waived the requirement for informed consent because only deidentified data were used. The study followed the Strengthening the Reporting of Observational Studies in Epidemiology (STROBE) reporting guideline.

The study used data from the American Heart Association Get With the Guidelines–Resuscitation (GWTG-R) registry, which contains detailed information on patients who experience cardiac arrest at participating hospitals in the United States. Within the GWTG-R registry, we identified all adults (aged ≥18 years) who developed IHCA during March to December 2020. Race and ethnicity were self-reported by the study patients, and these data were collected in the GWTG-R registry to examine disparities in care and outcomes of IHCA patients. We constructed multivariable hierarchical regression models to compare survival to discharge and return of spontaneous circulation (ROSC) for 20 minutes or more among patients with and without a suspected or confirmed COVID-19 infection. These models included hospital site as a random intercept and patient variables and calendar month as fixed effects. We used a Poisson link to directly estimate rate ratios. Data are presented as relative risks (RRs) with 95% CIs. Details on the GWTG-R registry, study cohort, study variables, and statistical analyses are included in the eMethods in the Supplement. All statistical analysis was performed in SAS version 9.4 (SAS Institute).

## Results

This study included 24 915 patients with IHCA from 286 hospitals who had a mean (SD) age of 64.7 (15.2) years. There were 9848 women (39.5%) and 15 066 men (60.5%), with sex missing for 1 patient. In terms of race and ethnicity, 6170 patients (24.8%) were Black, 15 223 (61.1%) were White, 949 (3.8%) were of other race or ethnicity (American Indian or Alaska Native, Asian or Pacific Islander, and other races and ethnicities), and 2573 (10.3%) were of unkown race or ethnicity. A suspected or confirmed COVID-19 infection was present in 5916 patients (23.7%). Patients with COVID-19 were younger, more frequently men and of Black race, and more likely to have an initial nonshockable rhythm, pneumonia, respiratory insufficiency, or sepsis and be receiving mechanical ventilation and vasopressors at the time of IHCA (Table 1). Patients with COVID-19 and IHCA had lower rates of survival to discharge (11.9% vs 23.5%; adjusted RR, 0.65 [95% CI, 0.60-0.71]; *P* < .001) and ROSC (53.7% vs 63.6%; adjusted RR, 0.86 [95% CI, 0.83-0.90]; *P* < .001). They were also more likely to have received delayed defibrillation (27.7% vs 36.6%; RR, 1.30 [95% CI, 1.09-1.55]; *P* = .003) but not delayed epinephrine treatment. The association between COVID-19 infection and worse survival outcomes was consistent for patients with nonsurgical diagnoses, patients in the intensive care unit (ICU), and patients who had received timely defibrillation or epinephrine treatment (Table 2).

**Table 1.** Baseline Characteristics of Patients With In-Hospital Cardiac Arrest, Overall and Stratified by the Presence or Absence of a Suspected or Confirmed COVID-19 Infection

Characteristic	Patients, No. (%)			*P* value
	Overall (N = 24 915)	With COVID-19 (n = 5916)	Without COVID-19 (n = 18 999)	
Demographic				
Age, y				
18-54	5576 (22.4)	1217 (20.6)	4359 (22.9)	<.001
55-64	5539 (22.2)	1341 (22.7)	4198 (22.1)	
65-74	6882 (27.6)	1765 (29.8)	5117 (26.9)	
75-84	4971 (20.0)	1210 (20.5)	3761 (19.8)	
≥85	1947 (7.8)	383 (6.5)	1564 (8.2)	
Sex				
Men	15 066 (60.5)	3778 (63.9)	11 288 (59.4)	<.001
Women	9848 (39.5)	2137 (36.1)	7711 (40.6)	
Missing	1	1	0	
Race and ethnicity				
Black	6170 (24.8)	1731 (29.3)	4439 (23.4)	<.001
White	15 223 (61.1)	3021 (51.1)	12 202 (64.2)	
Other^a^	949 (3.8)	256 (4.3)	693 (3.7)	
Unknown	2573 (10.3)	908 (15.4)	1665 (8.8)	
Cardiac arrest factor				
Illness category				
Medical noncardiac	13 106 (52.6)	4400 (74.4)	8706 (45.8)	<.001
Medical cardiac	8100 (32.5)	1299 (22.0)	6801 (35.8)	
Surgical noncardiac	1794 (7.2)	114 (1.9)	1680 (8.8)	
Surgical cardiac	1034 (4.2)	46 (0.8)	988 (5.2)	
Other	875 (3.5)	56 (1.0)	819 (4.3)	
Location of cardiac arrest				
ICU	11 746 (47.2)	3608 (61.0)	8138 (42.8)	<.001
Telemetry unit	3456 (13.9)	787 (13.3)	2669 (14.1)	
Nonmonitored hospital unit	3981 (16.0)	802 (13.6)	3179 (16.7)	
ED	3672 (14.7)	537 (9.1)	3135 (16.5)	
Procedural area	1627 (6.5)	125 (2.1)	1502 (7.9)	
Other	431 (1.7)	57 (1.0)	374 (2.0)	
Time of arrest				
Night (11 pm to 6:59 am)	7310 (29.6)	1750 (29.8)	5560 (29.5)	.67
Weekend	7767 (31.2)	1884 (31.9)	5883 (31.0)	.20
Initial cardiac arrest rhythm				
Pulseless electrical activity	14 646 (58.8)	3560 (60.2)	11 086 (58.4)	<.001
Asystole	6691 (26.9)	1795 (30.3)	4896 (25.6)	
Ventricular fibrillation	1843 (7.4)	252 (4.3)	1591 (8.4)	
Pulseless ventricular tachycardia	1735 (7.0)	309 (5.2)	1426 (7.5)	
Preexisting condition				
Heart failure this admission	2850 (11.4)	480 (8.1)	2370 (12.5)	<.001
History of heart failure	5722 (23.0)	1074 (18.2)	4648 (24.5)	<.001
Myocardial infarction this admission	3073 (12.3)	460 (7.8)	2613 (13.8)	<.001
History of myocardial infarction	3455 (13.9)	599 (10.1)	2856 (15.0)	<.001
Hypotension	8024 (32.2)	1936 (32.7)	6088 (32.0)	.33
Respiratory insufficiency	13 420 (53.9)	4020 (68.0)	9400 (49.5)	<.001
Renal insufficiency	9308 (37.4)	2439 (41.2)	6869 (36.2)	<.001
Hepatic insufficiency	2606 (10.5)	539 (9.1)	2067 (10.9)	<.001
Metabolic or electrolyte abnormality	7684 (30.8)	1961 (33.2)	5723 (30.1)	<.001
Diabetes	9377 (37.6)	2616 (44.2)	6761 (35.6)	<.001
Baseline depression in CNS function	1913 (7.7)	464 (7.8)	1449 (7.6)	.59
Acute stroke	939 (3.8)	159 (2.7)	780 (4.1)	<.001
Acute CNS nonstroke event	3867 (15.5)	825 (14.0)	3042 (16.0)	<.001
Pneumonia	5909 (23.7)	3044 (51.5)	2865 (15.1)	<.001
Major trauma	1197 (4.8)	174 (2.9)	1023 (5.4)	<.001
Sepsis	6020 (24.2)	1925 (32.5)	4095 (21.6)	<.001
Metastatic or hematologic malignant neoplasm	2715 (10.9)	378 (6.4)	2337 (12.3)	<.001
Intervention in place at time of cardiac arrest				
Continuous intravenous vasopressor	7937 (31.9)	2319 (39.2)	5618 (29.6)	<.001
Mechanical ventilation	10 677 (42.9)	3412 (57.7)	7265 (38.2)	<.001
Hemodialysis	980 (3.9)	280 (4.7)	700 (3.7)	<.001
Survival outcome				
ROSC	15 252 (61.2)	3176 (53.7)	12 076 (63.6)	<.001
Survival to discharge	5161 (20.7)	706 (11.9)	4455 (23.5)	<.001

Abbreviations: CNS, central nervous system; ED, emergency department; ICU, intensive care unit; ROSC, return of spontaneous circulation.

^a^ Includes American Indian or Alaska Native, Asian or Pacific Islander, and other races and ethnicities, which were combined for descriptive purposes.

**Table 2.** Association Between COVID-19 Infection and Survival Outcomes Among Patients With In-Hospital Cardiac Arrest

Characteristic	No. of patients	Adjusted RR (95% CI)	*P* value
Overall cohort	24 915		
Survival to discharge		0.65 (0.60-0.71)	<.001
ROSC		0.86 (0.83-0.90)	<.001
Medical patients only	21 206		
Survival to discharge		0.65 (0.59-0.71)	<.001
ROSC		0.86 (0.82-0.90)	<.001
ICU patients only	11 746		
Survival to discharge		0.66 (0.58-0.75)	<.001
ROSC		0.87 (0.82-0.92)	<.001
ICU patients with pneumonia only	3543		
Survival to discharge		0.61 (0.50-0.74)	<.001
ROSC		0.85 (0.77-0.94)	.001
Patients with prompt treatment^a^	19 906		
Survival to discharge		0.64 (0.51-0.81)	<.001
ROSC		0.85 (0.73-0.99)	.03

Abbreviations: ICU, intensive care unit; ROSC, return of spontaneous circulation.

^a^ Denotes patients who received prompt defibrillation for 2 minutes or less for a shockable arrest or prompt epinephrine for 5 minutes or less for a nonshockable arrest.

## Discussion

In this cohort study, approximately 1 in 4 patients with IHCA identified in the GWTG-R registry during 2020 had a suspected or confirmed COVID-19 infection. This observation underscores the sizable effect of the pandemic on in-hospital resuscitation. Even after accounting for substantial differences between patients with and without COVID-19 infection, the disease was associated with a one-third lower rate of overall survival and was accompanied by a 30% increased rate of delayed defibrillation in shockable IHCA. Although delays in resuscitation, especially defibrillation, may have contributed to lower survival, the negative association of COVID-19 with survival in this study was consistent across subgroups, including patients who received timely treatment with defibrillation and epinephrine.

The absolute survival rate we observed for US patients with IHCA and COVID-19 (11.9%) was higher than the survival rates reported initially (0%,^1^ 2.9%,^2^ and 3.0%^3^), which likely represented the isolated experience of health systems overwhelmed early during the pandemic. Our findings are consistent with those of Hayek et al^5^ (12.0% survival in 400 patients with COVID-19 in 68 ICUs) and Mitchell et al^6^ (11.9% survival in 260 patients with COVID-19 at 11 hospitals) and extend the previous findings by adding a comparison group of patients without COVID and including non-ICU patients from a larger group of hospitals. We believe that these data will be relevant to health care providers and hospital administrators as the COVID-19 pandemic continues. Because IHCA survival among patients with COVID-19 in this study was not as poor as reported previously, we believe that COVID-19 infection alone should not be used as a criterion for withholding resuscitation care from hospitalized patients.

Our study has the following limitations. First, despite robust risk adjustment, confounding as a result of unmeasured variables cannot be excluded. Second, GWTG-R is a quality improvement registry, and our findings may not be representative of nonparticipating hospitals. Finally, it is possible that some patients suspected to have a COVID-19 infection were later confirmed to have a negative test result; however, this was expected to occur infrequently, as data were typically abstracted once COVID-19 results were known. As new variants emerge, future studies will be needed to assess the ongoing impact of COVID-19 infection on IHCA survival.

## Notes

Supplement.
eMethods.

eReferences
