# Supplementary material for: Association of COVID-19 Infection With Survival After In-Hospital Cardiac Arrest Among US Adults
Source: JAMA Netw Open. 2022 Mar 2;5(3):e220752. doi: 10.1001/jamanetworkopen.2022.0752 (PMC8892224; doi:10.1001/jamanetworkopen.2022.0752)
Supplement: Supplement. — eMethods. eReferences [file jamanetwopen-e220752-s001.pdf]

Supplementary Online Content

Girotra S, Chan ML, Starks MA, Churpek M, Chan PS; American Heart Association Get With the Guidelines–Resuscitation Investigators. Association of COVID-19 infection with survival after in-hospital cardiac arrest among US adults. *JAMA Netw Open*. 2022;5(3):e220752. doi:10.1001/jamanetworkopen.2022.0752

eMethods.
eReferences

This supplementary material has been provided by the authors to give readers additional information about their work.

© 2022 Girotra S et al. *JAMA Network Open*.

eMethods

Data Source

The study was conducted using data from the American Heart Association's Get with the Guidelines-Resuscitation (GWTG-R) registry - a large, prospective, multisite registry of IHCA in the United States. The design of this registry has been previously described.[1] Hospitals participating in the registry submit clinical information regarding the medical history, hospital care, and outcomes of consecutive patients hospitalized for cardiac arrest using an online, interactive case report form and Patient Management Tool™ (IQVIA, Parsippany, New Jersey).

All patients with a confirmed in-hospital cardiac arrest (IHCA), defined as an absence of a palpable central pulse, apnea, and unresponsiveness and without do-not resuscitate orders, are enrolled by trained personnel at participating hospitals. Multiple case-finding approaches are used including review of centralized collection of cardiac arrest flow sheets, routine checks of code cards, pharmacy tracer drug records, reviews of hospital paging system logs, and hospital billing charges for resuscitation medications. Hospital participation is voluntary with data collected using standardized "Utstein-style" definitions for all patient variables and outcomes to facilitate uniform reporting across hospitals.[2,3] To ensure accuracy and completeness of the data, rigorous training and certification process of the medical staff at participating hospitals along with use of standardized software with internal checks, and periodic re-abstractions and audits of collected data. The study is reported in accordance with the Strengthening the Reporting of Observational Studies in Epidemiology (STROBE) guidelines. [4]

Study Cohort

We identified 31,145 adult patients ($\geq$18 years) who underwent cardiopulmonary resuscitation for an index, pulseless IHCA during 2020. Patients with IHCA during the months of January and February (n=5551) were not included due to very few cases of hospitalized COVID-19 patients in the United States during those months. We also excluded 380 patients with missing data on survival and 299 patients from 32 hospitals with no IHCA in COVID-19 patients.

Study Variables & Outcomes.

The primary exposure was whether the patient was suspected or confirmed to have COVID-19 infection, which was ascertained using a new variable added to the GWTG-R data collection form at the start of the pandemic. The study outcomes were survival to discharge and return of spontaneous circulation (ROSC) for at least 20 minutes.

Patient-level data from GWTG-Resuscitation included *demographic* variables (age, sex, race [self-reported]), *pre-existing conditions* (current or prior myocardial infarction, current or prior heart failure, diabetes mellitus, renal insufficiency, hepatic insufficiency, respiratory insufficiency, pneumonia, sepsis, hypotension, acute stroke, baseline central nervous system depression, major trauma, metastatic or hematologic malignancy, metabolic or electrolyte abnormalities), *cardiac arrest characteristics* (initial rhythm [VF or pulseless VT], illness category [medical non-cardiac, medical cardiac,

© 2022 Girotra S et al. *JAMA Network Open*.

surgical non-cardiac and surgical cardiac and other], location of arrest [intensive care unit {ICU}, telemetry unit, ward without telemetry, operating room, procedural area, emergency room, and other], time of day [daytime: 7AM-11PM, night-time: 11PM-7AM] and calendar month of the arrest), interventions in place immediately prior to arrest (mechanical ventilation, dialysis and intravenous vasoactive drugs).

Statistical Analysis

We compared baseline characteristics between COVID-19+ and non-COVID patients using chi-square test for categorical variable or t-test for continuous variables. Next, we compared the rates of study outcomes in COVID-19+ and non-COVID patients. To evaluate the independent association of COVID-19 infection with survival outcomes, we constructed two-level (hospital and patient) hierarchical regression models. Such models account for clustering of patients within a hospital and avoid overestimation of significance of statistical associations. The hospital site was included as a random intercept and all patient variables listed in the *Study Variables & Outcomes* section above were included as a fixed effect. Since the study period was characterized by multiple periods when COVID-19 cases across the country were increasingly rapidly, we also included calendar month of the arrest as a fixed effect. We used a Poisson link to directly estimate rate ratios instead of odds ratios from these models. We also compared delays in defibrillation (>2 minutes from onset of shockable IHCA) and delays in epinephrine (>5 minutes from onset of non-shockable IHCA onset).

Similar models were used to examine the association of COVID-19 infection and survival outcomes within the following subgroups: 1) medical patients only 2) ICU patients 3) ICU patients with pneumonia and 4) patients who received timely treatment. Timely treatment was defined as receipt of defibrillation within 2 minutes of onset of IHCA for patients with an initial shockable rhythm, or receipt of epinephrine within 5 minutes of onset of IHCA for patients with an initial non-shockable rhythm.

Statistical analysis was performed in SAS (version 9.4, SAS Institute, Cary, NC).

© 2022 Girotra S et al. *JAMA Network Open*.

eReferences

1. Peberdy MA, Kaye W, Ornato JP, et al. Cardiopulmonary resuscitation of adults in the hospital: a report of 14720 cardiac arrests from the National Registry of Cardiopulmonary Resuscitation. *Resuscitation*. Sep 2003;58(3):297-308. doi:10.1016/s0300-9572(03)00215-6
2. Cummins RO, Chamberlain D, Hazinski MF, et al. Recommended guidelines for reviewing, reporting, and conducting research on in-hospital resuscitation: the in-hospital 'Utstein style'. American Heart Association. *Circulation*. Apr 15 1997;95(8):2213-39. doi:10.1161/01.cir.95.8.2213
3. Jacobs I, Nadkarni V, Bahr J, et al. Cardiac arrest and cardiopulmonary resuscitation outcome reports: update and simplification of the Utstein templates for resuscitation registries. A statement for healthcare professionals from a task force of the international liaison committee on resuscitation (American Heart Association, European Resuscitation Council, Australian Resuscitation Council, New Zealand Resuscitation Council, Heart and Stroke Foundation of Canada, InterAmerican Heart Foundation, Resuscitation Council of Southern Africa). *Resuscitation*. Dec 2004;63(3):233-49. doi:10.1016/j.resuscitation.2004.09.008
4. von Elm E, Altman DG, Egger M, et al. The Strengthening the Reporting of Observational Studies in Epidemiology (STROBE) statement: guidelines for reporting observational studies. *Lancet*. Oct 20 2007;370(9596):1453-7. doi:10.1016/S0140-6736(07)61602-X

© 2022 Girotra S et al. *JAMA Network Open*.
